# The Cd/Zn Axis: Emerging Concepts in Cellular Fate and Cytotoxicity

**DOI:** 10.3390/biom13020316

**Published:** 2023-02-07

**Authors:** Colleen Elsa Johns, Mrudula Gattu, Samuel Camilli, Apoorva Desaraju, Narasaiah Kolliputi, Lakshmi Galam

**Affiliations:** Division of Allergy and Immunology, Department of Internal Medicine, Morsani College of Medicine, University of South Florida, Tampa, FL 33612, USA

**Keywords:** cadmium, zinc, inflammation, cytokines, immune system

## Abstract

Cadmium (Cd) is a toxic and carcinogenic substance that is present in the natural environment. The underlying biomolecular mechanisms of Cd toxicity are not completely understood, and it continues to be a significant research target due to its impact on public health. The primary routes of exposure are through ingestion of contaminated food and water and inhalation. Cd’s long biological half-life of 10–30 years allows it to accumulate in the body, leading to organ dysfunction notably in the kidney, liver, bone, and lungs. Cd has similar biochemical characteristics to Zinc (Zn). It shares the import transporters, ZIP8 and ZIP14, to enter the cells. This competitive behavior can be observed in multiple instances throughout the progression of Cd toxicity. Future studies on the biochemical interactions of Cd and Zn will elucidate the potential protective effects of Zn supplementation in reducing the effects of Cd toxicity. In addition, research can be focused on discovering key proteins and effective pathways for Cd elimination that confer fewer adverse effects than current antioxidant therapies.

## 1. Introduction

**Figure 1:**
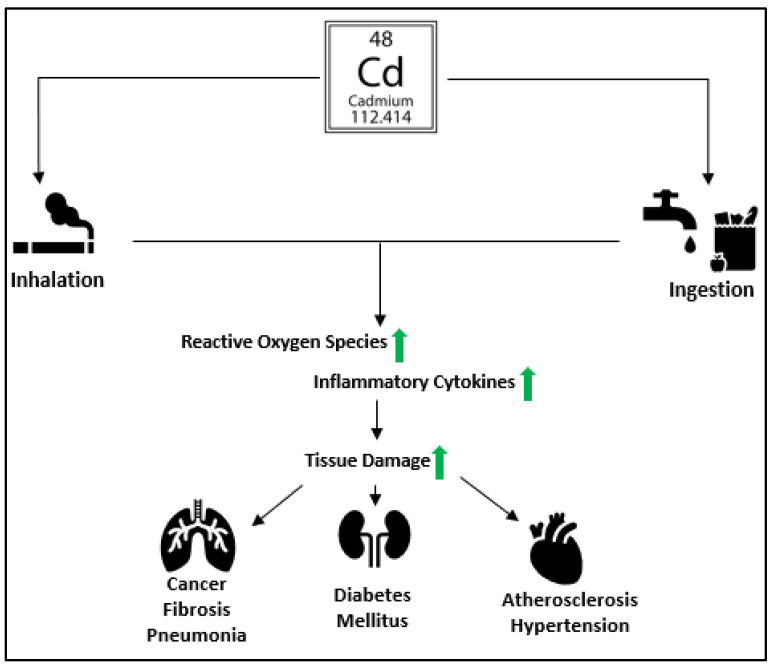
An illustration shows the effects of cadmium toxicity. Cadmium enters the human body from contaminated air, food, water, and tobacco smoking. It causes oxidative stress by increasing the production of ROS. Cd exposure results in the release of pro-inflammatory cytokines such as Interleukin-1 (IL-1), Interleukin-6 (IL-6), Interleukim-8 (IL-8), and Tumor necrosis factor-alpha (TNFα), leading to multi-organ tissue damage causing pneumonia, lung fibrosis, hypertension, diabetes mellitus, atherosclerosis, and cancer.

Cadmium (Cd) is a toxic heavy metal present in the natural environment. Sources of Cd include the paint and battery manufacturing industries, inhalation of polluted air, cigarette smoke, and oral ingestion of contaminated food and water (Figure 1) [1]. Plants absorb Cd from the soil and store it in high quantities in their leaves [2]. Among the sources of cadmium, oral ingestion and inhalation of Cd remain the primary routes of exposure [3]. Increased levels of Cd in foods are a significant public health concern for humans. For example, Itai-Itai disease, first recognized in Toyama Prefecture, Japan, resulted from chronic long-term oral Cd ingestion. The disease affects the kidneys, leading to renal tubular and glomerular dysfunction, Vitamin D insufficiency, and bone injury [4]. This paper examines the biochemical, cellular, and tissue-specific toxic effects of Cd. It also analyzes the prevailing theories on its mechanistic progression and the importance of exploring methods of detoxification to attenuate Cd-driven diseases.

## 2. Impacts on Immune Homeostasis

Furthermore, Cd disrupts hematopoiesis and affects the adaptive immune system by inhibiting the maturation of lymphocytes and altering their function in lymphoid tissues and the blood [5,6,7]. The International Agency for Research on Cancer (IARC) has categorized Cd as a Group I carcinogen [8]. The mechanisms by which Cd exerts its carcinogenicity include disrupting the DNA-repair system, deactivating tumor suppressor genes, and stimulating proto-oncogenes [9,10]. This disruption also promotes an exaggerated release of inflammatory cytokines [1,2,11]. These include IL-1, IL-6, IL-8, TNF-α, and Interferon-gamma (IFN-γ), and the anti-inflammatory cytokine, Interleukin-10 (IL-10) [1,12,13]. It is essential to note the differential effects of Cd on cytokine production. The increase in the number of allergic diseases and cancers seen in urban societies might be due to environmental contamination and the effect of Cd on the generation of cytokines and Immunoglobulin E (IgE) antibodies [14,15,16]. Several human health conditions, including cardiovascular diseases, are associated with inflammatory conditions caused by Cd exposure, as it leads to the intrusion of neutrophils into organs and stimulates the release of pro-and anti-inflammatory cytokines from monocytes/macrophages. 

These cell types are the crucial members of innate immunity and are most harmed by Cd. Although Cd can terminate immune activation in macrophages, immune activation in monocytes is stimulated by Cd. Activated monocytes further exacerbate inflammation, and defective macrophages are ineffective in producing an immune response. A survey of 8700 participants, published by the Third National Health and Nutrition Examination Survey (NHANES III), demonstrated that people who smoke have decreased dietary Zn, a high Cd load, and a high likelihood of developing Chronic Obstructive Pulmonary Disease (COPD). Respiratory infections in COPD patients aggravate the impaired macrophage function caused by Cd retention. These impaired macrophages can lead to a harmful cycle that worsens COPD due to the defective immune response [17]. Further investigation is needed to better understand the inflammatory pathways and immune mechanisms involved in Cd toxicity.

## 3. Oxidative Stress & Organelle Dysregulation

Chronic exposure to Cd results in the increased production of reactive oxygen species (ROS), increased lipid peroxidation, and a decrease in the protective antioxidant enzyme activity of superoxide dismutase (SOD) and catalase (CAT). Increased oxidative stress subsequent to Cd exposure is associated with health problems, including hypertension and insulin resistance [18,19]. Cd-mediated oxidative stress also affects the intensity and fatality associated with the novel, emerging respiratory infectious disease, Corona Virus Disease—2019 (COVID-19) infection. The respiratory system is impacted by a decreased respiratory function, a weakened ability to fight infections, and a chronic lung inflammatory state. Smoking is also associated with a higher risk of COVID-19 infections due to lung inflammation, diminished epithelial cell barrier activity, stimulation of matrix metalloproteinases, excessive mucous production from increased oxidative stress, and defective mucociliary clearance [20].

In addition to generating endoplasmic reticulum stress, Cd induces mitochondrial dysfunction. Branca et al. [21] established that retention of Cd in the proximal tubular epithelium of rat kidneys led to mitochondrial dysfunctions with elevated mitochondrial permeability, inflammation, and prevention of cellular respiration. These variations resulted in the development of ROS, culminating in renal cell apoptosis. A recent analysis of human renal proximal tubule (HK-2) cells exposed to Cd showed inhibition and reduced quality of mitochondrial Electron Transport Chain (ETC) complexes [22]. Previous evidence of Cd’s effect on the ETC revealed that concentrations as low as 1µM significantly reduce the transfer of electrons from the cytosol to the mitochondrial matrix of human bone cells [23]. Furthermore, Cd affects lysosomal activity. Lysosomes participate in the process of autophagy, an important process that involves the breakdown and removal of misfolded proteins and damaged organelles. Cd toxicity results in an inability of lysosomes to fuse with autophagosomes and causes defects in the function of lysosomes [24,25]. These alterations were found to be responsible for the neurotoxic effects induced by Cd in a murine neuro-crest cell line [26,27].

Another mechanism by which Cd exerts its toxicity in lung cells is through the upregulation of apoptosis. Activation of inflammatory pathways can lead to apoptosis of lung cells, resulting in fibrosis with decreased lung function [28]. Proapoptotic signals such as BCL2-associated X protein (Bax) and caspase 8 were stimulated, and anti-apoptotic B-cell lymphoma 2 (Bcl-2) signals were inhibited in lung cells that were exposed to Cd [20]. Future research should focus on the recognition of the mechanistic pathways that connect apoptosis and lung fibrosis.

## 4. Tissue Retention and Competitive Binding

Primarily, heavy metals such as zinc (Zn) and iron (Fe) are transported into the cell by their own uptake transporters. Divalent metal transporter 1 (DMT1) shuttles Fe across the cell membrane, and Zn is moved by ZIP8 and ZIP14. However, Cd’s similar biochemical features to Zn allow it to enter cells by using Zn transporters [17]. A study performed to test the affinity of the ZIP8 transporter demonstrated that the absorption of Cd was significantly reduced with the knockdown of ZIP8 transporters. The study concluded that the ZIP8 transport system is important in the absorption of Cd in kidney tubules. In mice, ZIP8 is present in the S3 segment of the proximal tubules of the kidney and functions in the reabsorption of Cd from the lumen to the epithelial cells [29]. The accumulation of Cd causes apoptosis or necrosis of epithelial cells. In this epithelial injury, the uptake of more Cd through the ZIP8 transporters will lead to increased inflammation. Thus, the ZIP8 transporter interaction with Cd has a significant influence on Cd-mediated toxicity [30,31].

In addition to having a long biological half-life of 10–30 years and dominant control on the flux of Zn transporters, Cd also has a reduced rate of excretion [3]. Metallothionein (MT), a low molecular weight protein, is rich in cysteine amino acids whose thiol groups allow metallothionein to bind to both Zn and Cd [32]. Current evidence suggests that Cd has a higher binding affinity to metallothionein than Zn, thus exhibiting another competitive relationship in which Cd competes with Zn for binding [33,34]. This higher binding affinity with metallothionein retains Cd in tissues. There are inadequate export pathways for Cd to be released from the body, and the target organs affected by Cd toxicity are the liver and kidneys [32]. Both the liver and kidneys are highly sensitive to Cd toxicity and accumulation, as these organs can produce metallothionein. The metallothionein will bind with Cd in the liver and form a CdMT complex that will travel through the circulatory system and be filtered and degraded by the kidneys [35]. The Occupational Safety and Health Administration defines a blood cadmium level of 5–10 µg/L of whole blood to be toxic, warranting medical removal from occupational exposure [36].

## 5. Oxidative Damage Therapies

Though Cd increases ROS and inflammation in cells and stimulates mitochondrial dysfunction and apoptosis, there are current therapies for detoxification. The most common therapeutic strategy for heavy metal toxicity is chelation therapy. Chelators are polydentate (multiple-bonded) ligands that form two or more coordinate bonds with heavy metals [37]. Ethylenediaminetetraacetic acid (EDTA) is used to treat Cd toxicity as well as lead poisoning, neurotoxicity, and atherosclerosis. Unfortunately, an array of adverse effects are associated with EDTA chelation therapy. The most common is renal toxicity, manifesting as acute tubular necrosis, renal failure, anuria, and proteinuria [38]. Interestingly, EDTA therapy is another point of interaction for Cd and Zn. Due to a high binding affinity for Zn, calcium EDTA can cause Zn deficiency [38]. These critical issues have caused great interest in understanding the affected biochemical pathways because of chelation therapy. Alternatives that confer reduced adverse effects and do not disrupt the levels of important elements in the body (Zn) are the focus of current efforts.

One such alternative to ameliorate the oxidative stress induced by Cd toxicity is vitamin E (VE). This trace element reduces free radicals [39,40]. Current studies on VE supplementation to reduce Cd-induced oxidative damage consider the exposure method and time of Cd administration, as well as the duration of VE [41,42]. Histopathological observation in one study saw increased swelling of the glomerulus and narrowed capsular space in the renal cortex and medulla of Cd-treated mice. However, the VE supplementation with Cd exposure greatly reduced the histopathological scoring of the same morphological changes observed in the Cd-only group [42]. Recent reports have taken this relationship between VE and Cd to investigate the antioxidative effects of other supplements [43,44,45]. One study used glimepiride, a common antidiabetic medicine, on Cd-treated mice. They observed a significant reduction of serum creatine and urea levels in the kidneys of the Cd/glimepiride group when compared to the Cd group [43].

## 6. Zn and Cd

We have previously mentioned a few occurrences of Zn and Cd sharing binding sites and pathway interactions, but there are a few more of note. Zn has an antagonizing role in the toxic effects of heavy metals, such as Cd. For instance, the addition of Zn to Cd-exposed bovine aorta endothelial cells caused a 1.1–2.0 fold reduction of intracellular ROS and a 1.1–2.0 fold reduction in apoptotic factors [46]. Studies also reveal that Zn supplementation correlates to increased activity of both B and T cells. In a study that attempted to display the effectiveness of Zn supplementation to restore immunity in Cd-exposed rats, splenic sections of rats that were treated with Cd resulted in significantly reduced B and T cells compared with Cd-treated sections supplemented with Zn [47]. Although the direct mechanism is not entirely understood, Zn can suppress the level of Cd toxicity. Pan et al. reported that adding Zn to Cd-exposed yeast cells effectively reversed the level of Cd differentially expressed genes’ DEGs [48]. Cd damages the signaling pathways which leads to malfunctioning in cells. These signaling pathways include the mitogen-activated protein kinase (MAPK), extracellular-signal-regulated kinase (ERK1/2), and c-Jun N-terminal kinase (JNK) pathways [17]. Another interaction occurs in the activity of estrogen receptors, in which two specialized protein motifs, zinc fingers, regulate the transcriptional activity of the receptor [49]. These motifs present another competitive binding site for Zn and Cd. Recent studies have implicated Cadmium in the activation of estrogen receptors simultaneously with its accumulation in tissues and oxidative damage [50,51]. Future investigation is necessary to elucidate the mechanisms of the signaling pathways involved and understand the critical points at which Cd and Zn interact.

Additionally, since Zn functions as a cytoprotectant and can maintain cellular homeostasis, intracellular Zn levels play an essential role in the cellular response after Cd uptake. The relationship is so direct that Zn deficiency escalates the absorption and retention of Cd [52]. The level of extracellular Zn present relative to Cd determines the effectiveness of Zn binding to ZIP8 instead of Cd, and this binding can determine the overall cellular response. However, Cd saturates these transporters with accumulation, causing a cycle of cellular dysfunction [29]. It is not completely understood whether supplemented Zn could directly increase the cellular export of Cd. It is essential to calculate the concentration of intracellular Zn that would be required to replace Cd-form complexes with proteins. However, it must also be noted that high amounts of either can be dangerous. In the intestines, a high Zn load causes the accumulation of the high zinc nuclear receptor (HIZR-1) protein [53]. The report used *Caenorhabditis elegans* to reveal that Cd also binds to HIZR-1. Interestingly, Cd in this system activates the high zinc response and upregulates transcripts that are involved in both Zn and Cd detoxification [53]. As a result, it seems that instead of decreasing protein function, Cd binding to HIZR-1 may cause aberrant activation in a situation where zinc is not in high concentration [53]. The ability of Cd to replace Zn as a ligand has been observed in multiple systems, but Cd binding HIZR-1 is unique in that it activates activity and transcription rather than inhibiting or blocking the function of Zn in the system.

Zn supports the function of both the innate and adaptive immune systems. Cd elevates Zn deficiency and dysregulates the immune system. Chronic exposure to Cd results in the collection of Cd inside the cells involved in the innate immune system. Cd tends to bind to the protein domain region and prevents vital metals from occupying the region and function. The immune system’s role is then modified, which results in the development of dangerous inflammatory responses leading to damage in the lungs, kidneys, and bones. When the function of the immune system is modified, the dose of Cd and cell type can regulate the activity of inflammatory responses.

## 7. Zn Is a Promising Therapeutic

Zinc is known to be protective against viruses, especially in COVID-19. Zn inhibits the activity of severe acute respiratory syndrome-associated coronavirus (SARS-CoV) RNA polymerase and reduces the activity of angiotensin-converting enzyme 2 (ACE2), a receptor for severe acute respiratory syndrome-associated coronavirus 2 (SARS-CoV-2). It hinders the nuclear factor kappa-light-chain-enhancer of activated B cells (NF-κB) signaling and curbs the cytokine surge in COVID-19. In addition, it enhances the mucociliary clearance and barrier function of the respiratory epithelium, leading to decreased probability of getting bacterial infections. One of the mechanisms by which Cd injures the lung is by competing with the Zn ion involved in respiratory defense [54]. Studies have shown a correlation between lower levels of zinc and a rise in pro-inflammatory TNFa expression and lung tissue alterations in rats. Lower levels of Zn can partly be restored by Zn supplementation. A deficit in Zn disrupts the lung epithelial cell barrier function by increasing TNFα, IFNy, and Fas receptor (FasR) signaling and cellular apoptosis. The inflammatory modifications of the lung extracellular matrix seen in pulmonary fibrosis are due to inadequate levels of Zn. Studies have shown that Zn pre-treatment minimizes acute lung injury in mice by reducing the mobilization of neutrophils driven by lipopolysaccharide (LPS) to the lungs. In the model of toxic environmental pollutant exposure, when animals with inadequate levels of Zn were exposed to hog barn dust rich in endotoxin, they experienced an increase in neutrophil shift and an increase in the production of pro-inflammatory cytokines such as TNFα, IL-6, and C-X-C Motif Chemokine Ligand 1 (CXCL1) [55]. Further clinical and experimental studies are necessary to discover the concentrations of Zn required to function as a preventative therapy for Cd-induced toxicity and respiratory infections such as COVID-19 [54].

## 8. An Emerging Concept of Cellular Fate

Our current understanding of programmed cell death involves three canonical pathways: apoptosis, pyroptosis, and necroptosis. Many protein families such as caspases and gasdermins are involved in these pathways, and during inflammatory cell death (pyroptosis), there is an assembly of the NOD-like receptor protein 3 inflammasome [56]. For many years, these pathways have been distinguished from one another, but there is an emerging concept that combines all three pathways into one comprehensive cell death scaffold. This new concept is called PANoptosis, and it has the potential to drastically change the scientific approach to cell death [57,58]. The simultaneous activation of multiple cell death pathways could have significant effects on targeted therapies. Rather than looking for an important regulator of one pathway, scientists can aim at an upstream regulator of all three pathways and prevent the activation entirely. This upstream regulator is known as the PANoptosome which acts as the molecular platform that activates each pathway. Many different knockout mice were used to determine which proteins were integral to the formation of the PANoptosome and subsequent cell death [59]. Cadmium toxicity is known to activate necroptosis [13], pyroptosis [60], and apoptosis [61]; however, no research has been done to determine if cadmium regulates this PANoptosome complex. These studies are only in animal models as well, further limiting clinical application of cadmium research.

## 9. Future Directions

Cd is an environmental pollutant that causes rampant oxidative stress and inflammation in multiple organ systems. Chronic exposure causes the dysregulation of innate immune mechanisms and a high tissue burden. Environmental pollution continues to become a worsening public health concern, making it necessary to improve human well-being. Current therapies such as chelators have adverse effects that create safety and efficacy concerns, thus creating a need for different perspectives. Future studies on the molecular, biochemical, and system-specific effects of Cd would help in the development of drug targets and may lead to better treatments in patients with Cd-induced organ dysfunction. PANoptosis offers a novel approach to the mechanistic understanding of cell death. By investigating these pathways as highly interconnected, researchers may find more promising therapeutic targets. When inhibited, these targets could entirely halt the progression of cell death and allow the body’s innate immune response to work effectively.

Additionally, the biochemical mechanisms underlying the association between Zn and Cd are not completely understood and there are still gaps in the quantification and explanation of Cd/Zn ratios. The competitive nature of these two metals and the disruption of normal Zn function creates a very important axis for the investigation of Cd toxicity. These specific points of competition such as ZIP8, HIZR-1, and metallothioneins are all great potential therapeutic targets and could vastly expand our mechanistic understanding of how Cd affects the body. Future studies should focus more on the potential benefits of Zn supplementation in response to early Cd exposure, before the accumulation and negative effects of Cd become irreversible. Combining research that investigates the Cd/Zn axis with investigations of Cd in PANoptosis offers an entirely new field to view heavy metal toxicity. Cadmium accumulates in tissues similarly to other heavy metals, but this does not mean that therapies for lead or copper toxicity will work for Cd, and they do not. A more comprehensive identification of Cd’s effects on cellular function is required to better understand the larger symptomology that is a result. PANoptosis offers greater potential for effective therapies, and identifying the specific points of contention for Cd and Zn could further this potential. Cellular dynamics is a field that requires more comprehensive analysis, as nearly all cellular processes are interconnected.

## Data Availability

No new data were created or analyzed in this study. Data sharing is not applicable to this article.
